# Dispositional mindfulness is associated with lower smartphone addiction through digital life balance among Chinese university students

**DOI:** 10.3389/fpsyg.2025.1653620

**Published:** 2025-10-16

**Authors:** Aamer Aldbyani, Guiyun Wang, Zhang Chuanxia, Afnan Alhimaidi

**Affiliations:** 1 Shandong Xiehe University, Jinan, China; 2 Princess Nourah bint Abdulrahman University, Riyadh, Saudi Arabia

**Keywords:** dispositional mindfulness, smartphone addiction, digital life balance, mindfulness, cross-sectional study

## Abstract

**Background:**

Prior research links mindfulness to lower levels of several addictive behaviors; however, the mechanisms for smartphone use remain less clear.

**Method:**

This cross-sectional study examined the association between dispositional mindfulness and smartphone addiction and tested the mediating role of digital life balance among 1,241 Chinese university students. Participants completed validated Chinese versions of the MAAS, SAS, and DLBS.

**Results:**

Correlation analyses showed that dispositional mindfulness was negatively associated with smartphone addiction (*r* = –0.41, *p* < 0.05) and positively associated with digital life balance (*r* = 0.45, *p* < 0.05). Mediation analysis using PROCESS (Model 4, 5,000 bootstrap samples) indicated a significant indirect effect of dispositional mindfulness on smartphone addiction through digital life balance (indirect effect = −0.18, SE = 0.019, 95% CI [−0.22, −0.14], while the direct effect remained significant (*β* = –0.23, *p* < 0.05).

**Discussion:**

These results indicate an indirect association in cross-sectional data, consistent with the possibility that digital life balance partly explains the link. The study provides evidence regarding the psychological processes linking dispositional mindfulness and technology use, suggesting potential applicability of promoting digital life balance as a strategy for addressing smartphone overuse.

## Introduction

Smartphones have become an essential component of university students’ daily lives, offering diverse academic and social functions that support learning, communication, and entertainment (Kumban et al., 2025). However, concerns have grown regarding excessive smartphone use—commonly referred to as “smartphone addiction”—which has been linked to disruptions in academic performance, social relationships, and overall psychological well-being (Al-Barashdi et al., 2015; Elamin et al., 2024).

Dispositional mindfulness refers to a relatively stable tendency to maintain nonjudgmental awareness of present-moment experiences (Brown et al., 2007). Mindfulness enables individuals to live with heightened awareness and clarity in the present, unburdened by preoccupations with the past or anxieties about the future, which has been associated with adaptive self-regulation (Alidina, 2020). Evidence supports the beneficial relationship between mindfulness and technology use: Liu et al. (2018) identified a negative correlation between mindfulness and excessive mobile phone use, while Arpaci et al. (2019) found that higher levels of mindfulness are linked to reduced symptoms of nomophobia.

Although dispositional mindfulness has been shown to reduce maladaptive technology use (Van Gordon, 2017), the pathways through which mindfulness exerts its protective effects remain insufficiently explored. Investigating digital life balance as a mediating factor provides evidence regarding how students can better manage their technology use in ways that promote well-being. Thus, this study seeks to address a significant gap in the literature by clarifying the role of digital life balance in the relationship between mindfulness and smartphone addiction, with implications for developing more targeted interventions aimed at fostering healthier digital habits among university students.

Accordingly, the present study examines whether digital life balance mediates the relationship between dispositional mindfulness and smartphone addiction among university students.

### Dispositional mindfulness and smartphone addiction

The use of smartphones has become an essential aspect of daily life for university students, serving a wide array of functions including internet access, academic engagement, peer communication, and entertainment activities such as gaming and social media use. Moreover, smartphones contribute significantly to academic success by providing access to digital libraries, research databases, and collaborative platforms, thereby enhancing the overall learning experience (Kumban et al., 2025). Despite these considerable benefits, growing concerns have emerged regarding the pervasive use of smartphones, particularly when usage escalates to problematic or addictive levels (Al-Barashdi et al., 2015).

Smartphone addiction, often classified as a non-substance behavioral addiction, is characterized by compulsive usage patterns that disrupt essential aspects of daily functioning, including interpersonal relationships and academic performance (Elamin et al., 2024). University students appear especially susceptible to this issue, given the dual role smartphones play in supporting both academic activities and social interactions (Abed et al., 2017; Achangwa et al., 2022). While smartphones offer considerable advantages, excessive use has been linked to a range of adverse psychological, social, and physical outcomes.

In this context, dispositional mindfulness has gained increasing attention as a potential protective factor. According to Kabat-Zinn (2003), dispositional mindfulness can be considered as a trait-like tendency to sustain nonjudgmental awareness of present experiences. Mindfulness, whether developed through formal training or naturally present to varying degrees across individuals, involves consciously attending to one’s current experiences without evaluation (Brown et al., 2007). However, Randal et al. (2015) further conceptualizes mindfulness as both a set of cultivable skills and a stable personality trait, emphasizing that individuals can strengthen their attentional focus through regular mindfulness practice. This practice promotes the ability to remain present without judgment, fostering greater psychological flexibility (Garland and Howard, 2013).

Mindfulness entails directing attention to current experiences rather than ruminating on past events or anticipating future outcomes. It involves intentional perception of thoughts and emotions without evaluating them as good or bad. Higher mindfulness has been linked to adaptive emotion regulation and attentional control, constructs relevant to technology use. Research by Burgoon et al. (2000) and Feltman et al. (2009) suggests that heightened mindfulness is associated with healthier lifestyles and the development of enhanced personal competencies.

An expanding body of literature has highlighted the potential of mindfulness in mitigating smartphone addiction (Arpaci, 2021; Chen et al., 2021; Felix et al., 2023; Kwon et al., 2013). Consistent findings through these studies indicate a robust inverse relationship between dispositional mindfulness and problematic smartphone use. Therefore, the present study proposes the following hypothesis:

*H*1: Dispositional mindfulness is negatively correlated with smartphone addiction.

### Digital life balance as a mediator

Maintaining a balanced digital life involves regulating technology use to support healthy routines and sustain meaningful social interaction, thereby contributing to overall well-being. Digital life balance (DLB) has recently evolved as a multidimensional construct reflecting the capacity to integrate digital technologies into daily routines in a way that supports, rather than undermines, psychological, social, and physical well-being. Early conceptualizations emphasized the harmonious management of online and offline activities (Duradoni et al., 2022), but subsequent research has expanded this view by highlighting its psychometric properties and cross-cultural applicability. Recent validation studies have refined the measurement of DLB and confirmed its structural invariance across diverse cultural contexts (Soysal et al., 2024; Lima-Costa et al., 2024; Malas et al., 2025; Tosti et al., 2025). These advances underscore DLB as not only a behavioral pattern but also an important indicator of adaptive digital engagement.

Moreover, emerging evidence links DLB to a range of psychological outcomes, including reductions in technology-related stress and improvements in subjective well-being, self-regulation, and social functioning (Duradoni et al., 2024; Duradoni et al., 2025). Prior DLB research provides a framework for understanding how it may mediate the link between mindfulness and smartphone addiction. Integrating these perspectives strengthens the rationale for our study and situates it within the contemporary discourse on digital well-being.

Although both digital life balance and smartphone addiction concern patterns of technology use, they capture distinct dimensions. Digital life balance reflects an adaptive capacity to regulate and integrate digital activities in ways that support overall well-being, while smartphone addiction denotes maladaptive, compulsive use that disrupts daily functioning. Validation studies of the Digital Life Balance Scale have provided evidence for its discriminant validity from measures of problematic smartphone use (Duradoni et al., 2022; Soysal et al., 2024; Lima-Costa et al., 2024), supporting the conceptual distinction between these two constructs.

Previous research has also explored alternative mediating pathways linking mindfulness to problematic smartphone use. For example, Kim et al. (2024) demonstrated that attentional impulsivity mediates the association between mindfulness and problematic smartphone use, suggesting that mindfulness may reduce excessive smartphone use in part by enhancing attentional control and reducing impulsive responses. Similarly, Liang et al. (2025) found that negative emotions and attentional control mediated the relationship between mindfulness and smartphone dependence among Chinese adolescents, indicating that mindfulness can influence smartphone use through its effects on emotional regulation and cognitive control. These findings highlight the need to consider multiple psychological pathways in understanding how mindfulness shapes technology-related behaviors and underscore the potential value of examining digital life balance as an additional mediating factor.

**Figure 1:**
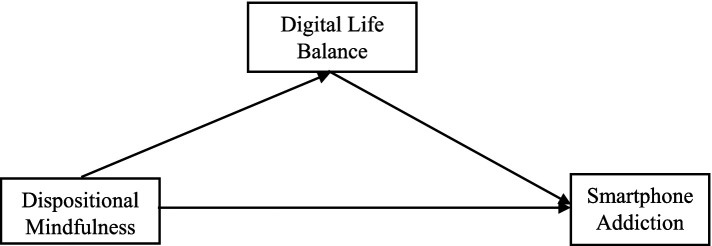
Study model.

To our knowledge, no empirical study has directly examined DLB as a mediator of the mindfulness–smartphone addiction association in this population. Additionally, based on self-regulation and attention-control theories, dispositional mindfulness may foster a more deliberate allocation of attention and time across online and offline domains, thereby facilitating adaptive digital life balance. This regulatory pathway provides a theoretically grounded rationale for considering digital life balance as a mediator between mindfulness and smartphone addiction (Figure 1). Therefore, the second hypothesis is as follows.

*H*2: Digital life balance mediates the relationship between dispositional mindfulness and smartphone addiction.

## Methods

A cross-sectional study was designed to determine if digital life balance mediates the relationship between dispositional mindfulness and smartphone addiction.

### Participants

The study sample comprised 1,241 undergraduate students (age range: 18–22 years; M = 21.12, SD = 1.36) from Shandong Xiehe University, China. All participants provided informed consent after being assured of the confidentiality and anonymity of their responses. The research protocol received approval from the university’s Academic Committee and was conducted in accordance with the ethical principles of the Declaration of Helsinki. Demographic characteristics of the participants are presented in Table 1.

**Table 1:** Demographic characteristics of the participants.

Variable	Frequency	%
Gender		
Male	499	40.2%
Female	742	59.8%
Family income		
3,000–9,999 Renminbi	741	59.71%
10,000–19,999 Renminbi	314	25.30%
≥ 20,000 Renminbi	186	14.99%
Total	1,241	100%

RMB, Renminbi.

### Measurements

#### Dispositional mindfulness

The 15-item Chinese version of the Mindful Attention Awareness Scale (MAAS; Brown and Ryan, 2003) was used to measure dispositional mindfulness. Responses were recorded on a 6-point Likert scale ranging from 1 (*Almost always*) to 6 (*Almost never*). Following the established scoring protocol, items are reverse-scored, with higher total scores indicating greater mindfulness. The validity of this version for Chinese contexts has been supported by Chen et al. (2012). In the present study, the MAAS demonstrated acceptable reliability (Cronbach’s *α* = 0.82).

#### Smartphone addiction

The Chinese short version of the Smartphone Addiction Scale (SAS-SV; Kwon et al., 2013), a 10-item instrument previously validated for use in China, was employed to measure smartphone addiction. Responses were captured on a 5-point Likert scale ranging from 1 (*Strongly disagree*) to 5 (*Strongly agree*). For the present study, the internal consistency of the SAS-SV was good, with a Cronbach’s alpha of 0.86.

#### Digital life balance

The Chinese version of the Digital Life Balance Scale (DLBS) used in this study was translated from the original English version (Duradoni et al., 2022) using established cross-cultural adaptation procedures. The DLBS comprises four items that assess an individual’s perceived ability to harmonize online and offline activities in daily life. Each item is rated on a 7-point Likert scale ranging from 1 (*strongly disagree*) to 7 (*strongly agree*). The translation was conducted collaboratively with bilingual colleagues from several Chinese universities. A back-translation procedure was undertaken to ensure conceptual equivalence, and the translated items were reviewed by a panel of experts in psychology and educational sciences. A pilot test was then conducted with a sample of university students (*n* = 72) to assess item clarity and cultural appropriateness. In the present study, the scale demonstrated good internal consistency (Cronbach’s *α* = 0.80), supporting its reliability in this context. All translated items are available from the authors upon request.

### Data analysis

Pearson correlations were calculated to assess the bivariate relationships between the variables. The hypothesized mediation was tested using the PROCESS macro (v. 3.5) in SPSS (Hayes, 2018). We employed a bootstrap approach with 5,000 resamples to generate a bias-corrected 95% confidence interval for evaluating the significance of the indirect effect.

## Results

### Correlation among study variables

As shown in Table 2, dispositional mindfulness was negatively correlated with smartphone addiction and positively correlated with digital life balance, which was itself negatively associated with addiction. All bivariate correlations were statistically significant.

**Table 2:** Correlation among the study variables (*n* = 1,241).

Variables	1	2	3
1. *MAAS*	1		
2. *SAS*	−0.41	1	
3. *DLBS*	0.45	−0.50	1
M	4.90	4.42	3.00
SD	1.80	1.62	0.72

*p* < 0.05. MAAS, Mindful Attention Awareness Scale; SAS, Smartphone Addiction Scale; DLBS, Digital Life Balance Scale.

### Mediation effects

A simple mediation analysis was conducted using Model 4 of the SPSS PROCESS macro (Hayes, 2018) with 5,000 bootstrap samples to test the mediating role of digital life balance in the relationship between dispositional mindfulness (independent variable) and smartphone addiction (dependent variable). After controlling for gender, age, and family income—which were not significant covariates—the results indicated a significant total effect of dispositional mindfulness on smartphone addiction (*β* = −0.41, *p* < 0.05). The direct effect remained significant (*β* = −0.23, *p* < 0.05), suggesting partial mediation. Detailed results are presented in Table 3.

**Table 3:** Path analysis.

Path	Coefficient (β)	Standard error (SE)	t-value (t)	Significance
a (MAAS → DLBS)	0.45	0.028	15.85	Significant
b (DLBS→ SAS)	−0.40	0.032	12.38	Significant
c (Total Effect)	−0.41	0.028	14.44	Significant
c’ (Direct Effect)	−0.23	0.030	−7.73	Significant
Indirect Effect (a*b)	−0.18	0.019	−9.37	Significant

*p* < 0.05. MAAS, Mindful Attention Awareness Scale; SAS, Smartphone Addiction Scale; DLBS, Digital Life Balance Scale.

**Figure 2:**
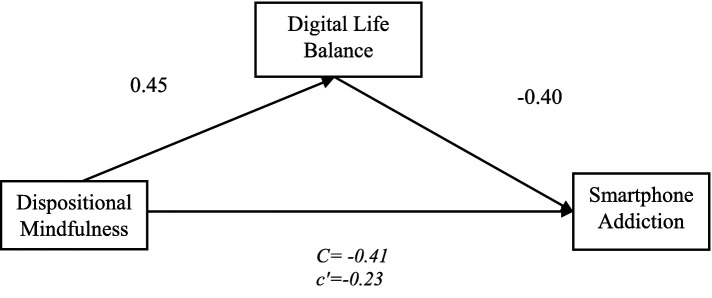
Mediation effects.

As summarized in Table 4 and Figure 2, the regression analyses indicated that dispositional mindfulness was a positive predictor of digital life balance (Model 1), which, in turn, negatively predicted smartphone addiction (Model 2). The mediation analysis, using the bias-corrected bootstrap method, confirmed a significant indirect effect (ab = −0.18, SE = 0.019, 95% CI [−0.22, −0.14]). This pattern of results supports partial mediation, suggesting that dispositional mindfulness reduces smartphone addiction both directly and indirectly by fostering a greater sense of digital life balance.

**Table 4:** Mediation effects.

Predictors	*Model 1 (DLBS)*		*Model 2 (SAS)*	
	*β*	*SE*	*β*	*SE*
MAAS	0.45	0.028	−0.23	0.030
DLBS			−0.40	0.032

*p* < 0.05. MAAS, Mindful Attention Awareness Scale; SAS, Smartphone Addiction Scale; DLBS, Digital Life Balance Scale.

## Discussion

The first objective of this study was to investigate the relationship between dispositional mindfulness and smartphone addiction in a sample of 1,241 Chinese undergraduates. The analysis revealed a significant negative correlation, indicating that higher mindfulness is associated with less severe addiction symptoms. This result aligns with existing literature documenting an inverse relationship between these constructs (Cheng et al., 2020; Jeong and Bae, 2024; Kayiş, 2022).

Additionally, the study revealed a positive correlation between dispositional mindfulness and digital life balance. This result partially aligns with earlier studies reporting that mindfulness is positively associated with engagement in digital detox behaviors (Achangwa et al., 2022; Aini et al., 2021; Remskar et al., 2024). The underlying mechanism for these associations may stem from mindfulness’s capacity to enhance digital self-regulation strategies, thereby mitigating the adverse effects of excessive smartphone use. Collectively, the findings are consistent with the view that mindfulness-based programs could support healthier technology use, pending confirmation in longitudinal or experimental studies. The persistence of a significant direct effect of dispositional mindfulness on smartphone addiction, even after accounting for digital life balance, indicates that this relationship is not fully explained by the mediator. This suggests that dispositional mindfulness may also reduce smartphone addiction through other mechanisms, such as enhanced emotional regulation, reduced impulsivity, or stronger self-control, which operate independently of digital life balance. Alternatively, it may point to a more fundamental direct association between mindfulness and smartphone addiction that does not depend on patterns of digital engagement. Future research should examine these potential pathways to provide a more comprehensive understanding of the processes linking mindfulness to smartphone use.

The study’s second objective, to investigate the mediating role of digital life balance, was supported. The results identified digital life balance as a significant partial mediator in the relationship between dispositional mindfulness and smartphone addiction. This indicates that while the cultivation of a balanced digital life is an important mechanism, it does not fully explain the direct negative impact of mindfulness on problematic use.

These findings are corroborated by theoretical frameworks in positive psychology, which posit that mindfulness fosters digital life balance through enhanced awareness, improved attention control, and better emotional regulation. The results demonstrate that by cultivating these capacities, mindfulness helps students achieve a healthier relationship with technology, thereby reducing smartphone addiction. This suggests that incorporating mindfulness-based interventions into university wellness programs could be a viable strategy for promoting digital wellbeing. While the present study conceptualized dispositional mindfulness as an antecedent variable, it can also serve as a moderator that influences how risk factors such as stress, impulsivity, or fear of missing out contribute to problematic smartphone use. For instance, Zhang and Wang (2022) demonstrated that trait mindfulness moderated the relationship between stress and problematic smartphone use, buffering its impact. Acknowledging this potential moderating role broadens the theoretical understanding of mindfulness in the context of digital behaviors and suggests that its protective influence may extend beyond a simple antecedent pathway.

Within the Psychology of Harmony framework, maladaptive digital engagement can be understood as compensation for unmet needs—especially control and relatedness—in offline contexts. Mindfulness may support digital life balance through two functions: it reduces the likelihood that stress generates need frustration and it strengthens self-regulation when such frustration occurs, limiting reliance on maladaptive online coping. This account clarifies how mindfulness could relate to digital balance in the proposed mediation model.

Overall, prior findings suggest that cultivating mindfulness meaningfully enhances digital-life balance, which in turn lowers the risk of problematic smartphone use in university populations. The findings suggest that incorporating brief, scalable mindfulness modules into orientation courses could be a promising approach to support fostering healthier technology habits and broader psychological well-being.

Like many studies based on self-report questionnaires, the present research is subject to potential biases that may affect the validity and generalizability of the findings. Self-reported data are vulnerable to distortions such as social desirability and recall bias, even though steps were taken to ensure anonymity and encourage honest responses. Future investigations should complement self-reports with behavioral and objective indicators, including digital tracking of smartphone use, ecological momentary assessments, and reports from peers or teachers, to improve measurement robustness and minimize reporting bias.

A further limitation is the restricted sample, which consisted solely of students from Shandong Xiehe University. This homogeneous sample limits the external validity of the results. Expanding future studies to include students from diverse academic disciplines, types of institutions, and cultural backgrounds would strengthen generalizability and enhance understanding of the phenomena across different populations.

The study assessed dispositional mindfulness exclusively with the MAAS, a widely used single-factor measure that does not capture the full multidimensional structure of mindfulness. Including additional multidimensional instruments in future research, together with key psychopathological covariates—particularly depressive symptoms and related psychological distress—would help clarify the unique contribution of mindfulness and reduce the risk of confounding. The well-documented comorbidity between smartphone addiction and depression (e.g., Zhang and Wang, 2024) means that depressive symptoms could be a significant confounding variable or an outcome in its own right.

Another important limitation concerns the cross-sectional design, which restricts the ability to draw causal inferences from the observed associations. Longitudinal or experimental designs are needed to test whether changes in mindfulness or digital life balance predict subsequent changes in smartphone addiction.

Beyond these limitations, future research should investigate whether dispositional mindfulness also acts as a moderator that buffers the impact of established risk factors such as perceived stress, attentional impulsivity, or negative affect on problematic smartphone use. Building on existing evidence (e.g., Zhang and Wang, 2022), studies could test models where mindfulness interacts with these risk factors to influence technology use. Examining mediation, preferably within longitudinal or experimental frameworks, would help delineate the boundary conditions of the current mediation model and offer a more comprehensive explanation of the role of mindfulness.

In addition, future work should address the documented comorbidity between smartphone addiction and depressive symptoms. Research has shown complex, heterogeneous patterns linking these variables (Zhang and Wang, 2024). Incorporating depressive symptoms into analytical models would allow researchers to test whether the protective role of mindfulness operates independently of, or partly through, reductions in such symptoms. This approach would deepen understanding of the psychological processes involved and inform the design of more targeted prevention and intervention strategies.

Finally, given the practical implications of the findings, future research should explore how mindfulness-based interventions can be adapted and implemented in educational and clinical settings to strengthen digital life balance and encourage healthier technology use. Such evidence would help bridge research and practice and guide policy-relevant programs aimed at reducing the risk of smartphone overuse.

## Conclusion

This study demonstrates that dispositional mindfulness serves as a protective factor against smartphone addiction among Chinese college students, with higher mindfulness levels associated with lower risk of problematic use. Furthermore, the findings reveal that mindfulness promotes digital life balance, which in turn reduces smartphone addiction. The identification of digital life balance as a partial mediator clarifies the mechanism through which mindfulness exerts its protective influence. These results provide empirical support for developing mindfulness-based interventions that specifically target digital life balance in educational settings.

## Data Availability

The raw data supporting the conclusions of this article will be made available by the authors, without undue reservation.
