# Correction: Dispositional mindfulness is associated with lower smartphone addiction through digital life balance among Chinese university students

**DOI:** 10.3389/fpsyg.2025.1730189

**Published:** 2025-11-19

**Authors:** Aamer Aldbyani, Guiyun Wang, Zhang Chuanxia, Afnan Alhimaidi

**Affiliations:** 1 Shandong Xiehe University, Jinan, China; 2 Princess Nourah bint Abdulrahman University, Riyadh, Saudi Arabia

**Keywords:** dispositional mindfulness, smartphone addiction, digital life balance, mindfulness, cross-sectional study

In the published article, an incorrect **Funding** statement was provided.

“The author(s) declare that no financial support was received for the research and/or publication of this article”.

The correct **Funding** statement reads:

“The author(s) declare that financial support was received for the research and/or publication of this article. The authors extend their appreciation to Princess Nourah bint Abdulrahman University (PNURSP2025R553) for their invaluable institutional support and participation in this research.”

A correction is also required to the **Acknowledgments** statement which was erroneously excluded from the article.

The correct **Acknowledgments** statement reads:

“The authors extend their appreciation to Princess Nourah bint Abdulrahman University (PNURSP2025R553) for their invaluable institutional support and participation in this research. Special thanks are due to Afnan Alhimaidi.”

The original version of this article has been updated.

